# High CENPA expression in papillary renal cell carcinoma tissues is associated with poor prognosis

**DOI:** 10.1186/s12894-022-01106-4

**Published:** 2022-09-26

**Authors:** Junwu Li, Qinke Li, Yang Yuan, Yiteng Xie, Yuanfeng Zhang, Ronggui Zhang

**Affiliations:** Department of Urology, The Second Affiliated Hospital of Chongqing Medical University, Chongqing, 400000 China

**Keywords:** CENPA, Papillary renal cell carcinoma, Prognostic analysis, Bioinformatics

## Abstract

**Objective:**

This work focused on investigating the relation of centromeric protein A (CENPA) gene expression with prognosis of papillary renal cell carcinoma (PRCC).

**Methods:**

We obtained data from PRCC cases in TCGA. Thereafter, CENPA levels between the paired PRCC and matched non-carcinoma samples were analyzed by Wilcoxon rank-sum test, while the relations of clinicopathological characteristics with CENPA level were examined by logistic regression and Wilcoxon rank-sum test. The prognostic value of CENPA was assessed by plotting the receiver operating feature curve (ROC) and calculating the value of area under curve (AUC). In addition, relations between clinicopathological characteristics and PRCC survival were analyzed through Kaplan–Meier (KM) and Cox regression analyses. After dividing the total number of patients into the trial cohort and the validation cohort in a ratio of 7:3, we constructed a nomogram in trial cohort according to multivariate Cox regression results for predicting how CENPA affected patient survival and used the calibration curve to verify its accuracy in both cohorts. We also determined CENPA levels within cancer and matched non-carcinoma samples through immunohistochemistry (IHC). Finally, we utilized functional enrichment for identifying key pathways related to differentially expressed genes (DEGs) between PRCC cases with CENPA up-regulation and down-regulation.

**Results:**

CENPA expression enhanced in PRCC tissues compared with healthy counterparts (*P* < 0.001). CENPA up-regulation was related to pathological TNM stage and clinical stage (*P* < 0.05). Meanwhile, the ROC curves indicated that CENPA had a remarkable diagnostic capacity for PRCC, and the expression of CENPA can significantly improve the predictive accuracy of pathological TNM stage and clinical stage for PRCC. As revealed by KM curves, PRCC cases with CENPA up-regulation were associated with poor survival compared with those with CENPA down-regulation (Risk ratio, RR = 3.07, 95% CI: 1.58–5.97, *P* = 0.001). In the meantime, univariate as well as multivariate analysis showed an independent association of CENPA with overall survival (OS, *P* < 0.05) and the nomogram demonstrated superior predictive ability in both cohorts. IHC analysis indicated that PRCC cases showed an increased CENPA positive rate compared with controls. As revealed by functional annotations, CENPA was enriched into pathways associated with neuroactive ligand receptor interactions, cytokine receptor interactions, extracellular matrix regulators, extracellular matrix glycoproteins and nuclear matrisome.

**Conclusion:**

CENPA expression increases within PRCC samples, which predicts dismal PRCC survival. CENPA may become a molecular prognostic marker and therapeutic target for PRCC patients.

## Introduction

PRCC is regarded as the second most common subtype of renal cancer, accounting for approximately 15–20% of the total renal cancer cases [1]. The underlying molecular mechanisms of PRCC, a heterogeneous disease, are not completely understood yet [2]. Meanwhile, the efficacy of targeted therapy in patients with advanced PRCC remains uncertain, although many recent studies are being conducted to explore the best risk gene characteristics in PRCC. To date, there is still a lack of adequate research on PRCC prognostic biomarkers. As a result, it is of crucial importance to identify new prognostic biomarkers and therapeutic targets to improve the survival rate of PRCC patients.

CENPA is a variant of the centromere-specific histone H3, which has a key role in centromere formation. It can transform the centromere into a complex consisting of a chromosome and a protein while ensuring the normal formation and function of the centromere and kinetomeres. In terms of cell-cycle regulation, cell division, and gene stability, the elevated CENPA gene expression can lead to CENPA mislocalization. Subsequently, the ectopic new centromeres and kinetomeres are formed in the chromosomal arms. These ectopic structures can then disrupt the normal segregation of chromosomes in cell division and aneuploidy formation, resulting in tumorigenesis. Alternatively, there is a direct link between the elevated CENPA gene expression and genomic instability, a condition that also triggers cancer occurrence and promotes disease progression. Numerous studies have identified the aberrant expression of CENPA gene in cancers, but related data in PRCC have not been reported yet.

The present work focused on illustrating the relation of CENPA with PRCC and analyzing CENPA’s effect on predicting PRCC prognosis according to TCGA-derived RNA-sequencing (RNA-seq) data. Besides, CENPA expression within PRCC as well as matched samples was analyzed, then, the relation of CENPA expression with overall OS of patients was analyzed. In addition, prognostic and clinical relevance studies were conducted for exploring the significance of CENPA in diagnosing and predicting patient prognosis. Moreover, enrichment, together with immune infiltration analyses were carried out to check the biological value of CENPA.

## Materials and methods

### RNA-seq data sources

Totally 321 PRCC cases with available gene expression data (KIRP-TPM) were gathered from TCGA database, while samples with RNA-seq data but without corresponding clinical data were excluded. Meanwhile, TPM data from 321 PRCC patients were further analyzed after filtering, and gene expression data were classified into low and high expression groups on the basis of the median CENPA expression level. This study complied with the publication guidelines formulated by the TCGA, and all the data used in this work were obtained from TCGA, so the patient ethical approval and informed consent were not needed.

### Statistical analysis

R (version 3.6.3) software was employed for performing statistical analysis. CENPA levels within tumor and non-carcinoma samples were explored by Wilcoxon rank-sum test. Besides, Kruskal–Wallis test, logistic regression and Wilcoxon rank-sum test were conducted to evaluate the relationship between clinicopathological characteristics and CENPA expression in logistic regression. By pROC package, we conducted ROC analysis and common binary assessment for assessing whether CENPA level was effective on separating PRCC cases from healthy subjects. After calculation, AUC values were 0.5–1, which indicated the 50%-100% discrimination ability. We also collected prognosis data, and prognostic factors were assessed based on KM and Cox regression analyses. *P* < 0.05 stood for statistical significance for each test.

### Construction and validation of the nomogram

After dividing the total number of patients into the trial cohort and the validation cohort in a ratio of 7:3, we established the nomogram in trial cohort by R software RMS package according to multivariate analysis results to predict 1-, 3- and 5-year survival of different individuals, which include clinicopathological characteristics that were significantly associated with CENPA. Calibration curve is the most commonly used method to evaluate model performance, which was assessed graphically through comparing our nomogram-estimated values with actual results in the present work, and 45°-line represented the optimal predicted results. Moreover, we also adopted concordance index (C-index) for assessing our nomogram discrimination. Then, in both corhorts, we estimated the prediction accuracy of our nomogram based on calibration curves.

### IHC analysis

We selected 4 PRCC patients treated in the Urology Department from January 2020 to October 2021, and collected both cancer and normal tissues as CENPA and control groups, respectively. The patient inclusion criteria were presented as follows, (1) patients confirmed with PRCC after paraffin specimen preparation; (2) patients that were diagnosed for the first time; and (3) patients who did not receive radiotherapy, chemotherapy or molecular targeted therapy in our hospital or other hospitals. The current work was approved by the Ethics Committee of our hospital and conducted following Declaration of Helsinki from the World Medical Association. All patients and their families signed the informed consents. The 4-um paraffin sections were washed by xylene and hydrated with gradient ethanol solution to water for deparaffinage and hydration. Later, the sections were boiled within sodium citrate buffer (0.01 mol/L, pH 6.0) to achieve antigen heat repair for a 20-min period. Thereafter, 3% hydrogen peroxide was added dropwise for inactivating tissue endogenous peroxidase. Then, sections were incubated at room temperature for 10 mins, washed by 1% bovine serum albumin (BSA), and incubated for a 30-min period under ambient temperature. After discarding the blocking solution, sections were incubated with primary antibody (1:100 dilution of CENPA antibody) at 4 °C overnight, washed with biotin-conjugated secondary antibody for a 40-min period under 37 °C, and washed with HRP-labeled streptomycin working solution. Afterwards, sections were stained with DAB and hematoxylin, dehydrated and transparentized with gradient ethanol solution and xylene, and sealed with neutral gum. Finally, photographs were taken under an optical microscope.

### Differential expression analysis

We compared gene levels (HTSeq-Counts) in CENPA up-regulation and down-regulation groups. Thereafter, by adopting Wilcoxon rank-sum test of DESeq2 software (3.8), we identified the DEGs between CENPA up-regulation and down-regulation groups on this basis. This study selected adjusted *P* < 0.05 and |log Fold change (FC)|> 1.5 to be the thresholds to identify DEGs. Those identified DEGs were later subject to Gene Ontology (GO) analysis with Clusterprofiler software 3.6.0.

### Gene enrichment analysis

This study adopted GSEA for determining the statistical significance of a gene set and the uniform difference between 2 biological statuses. We conducted GSEA with ClusterProfiler in R package for illustrating the significant differences in functions and pathways between CENPA up-regulation and down-regulation groups. Functions and pathways satisfying the thresholds of false discovery rate (FDR) < 0.25 and adjusted *P* < 0.05 were deemed to be significantly enriched.

### Immune infiltration analysis

CENPA samples were analyzed by ssGSEA with R software GSVA package. 24 immune cell types were analyzed, including mast cells, neutrophils, macrophages, eosinophils, CD56bright NK cells, CD56dim NK cells, natural killer (NK) cells, activated DC (aDC), dendritic cells (DC), immature DC (iDC), plasmacyte-like DC (pDC), CD8 + T cells, T cells, Th1/Th2/Th17 cells, T helper (Th) cells, regulatory T cells (Treg), T follicle helper cells, effector memory T cells (Tem), central memory T cells (Tcm), B cells, cytotoxic cells, and T cells (Tgd). We determined the enrichment score of every cell type based on gene levels within cancer samples and those reported key genes in 24 types of immune cells. Additionally, we conducted Spearman correlation for determining the relation of PRCC with immune cell subpopulations. In addition, Wilcoxon rank-sum test was adopted for investigating the immune cell infiltration levels in CENPA up-regulation and down-regulation groups.

## Results

### CENPA expression elevated in PRCC samples

**Fig. 1:**
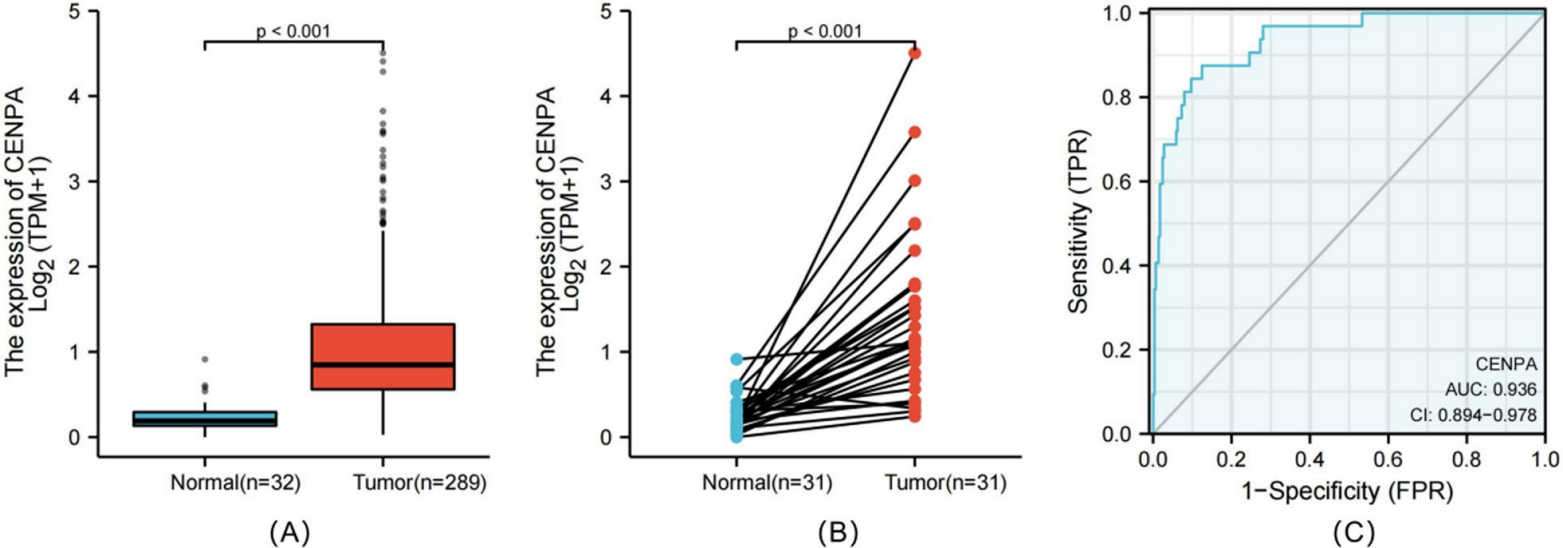
CENPA expression between cancer and normal tissues in PRCC patients (**A**). CENPA expression levels in PRCC and matched normal tissues (**B**). ROC analysis of CENPA shows promising discrimination power between tumor and normal tissues (**C**)

CENPA expression in 289 tumor tissues was substantially higher than that in 32 normal tissues (*P* < 0.001) (Fig. 1A), and it was also higher in 31 tumor tissues (*P* < 0.001) (Fig. 1B). Moreover, CENPA expression exhibited promising discrimination ability, with an AUC value of 0.936 in distinguishing tumors from normal tissues (Fig. 1C).

### Association between CENPA expression and clinicopathological characteristics

**Fig. 2:**
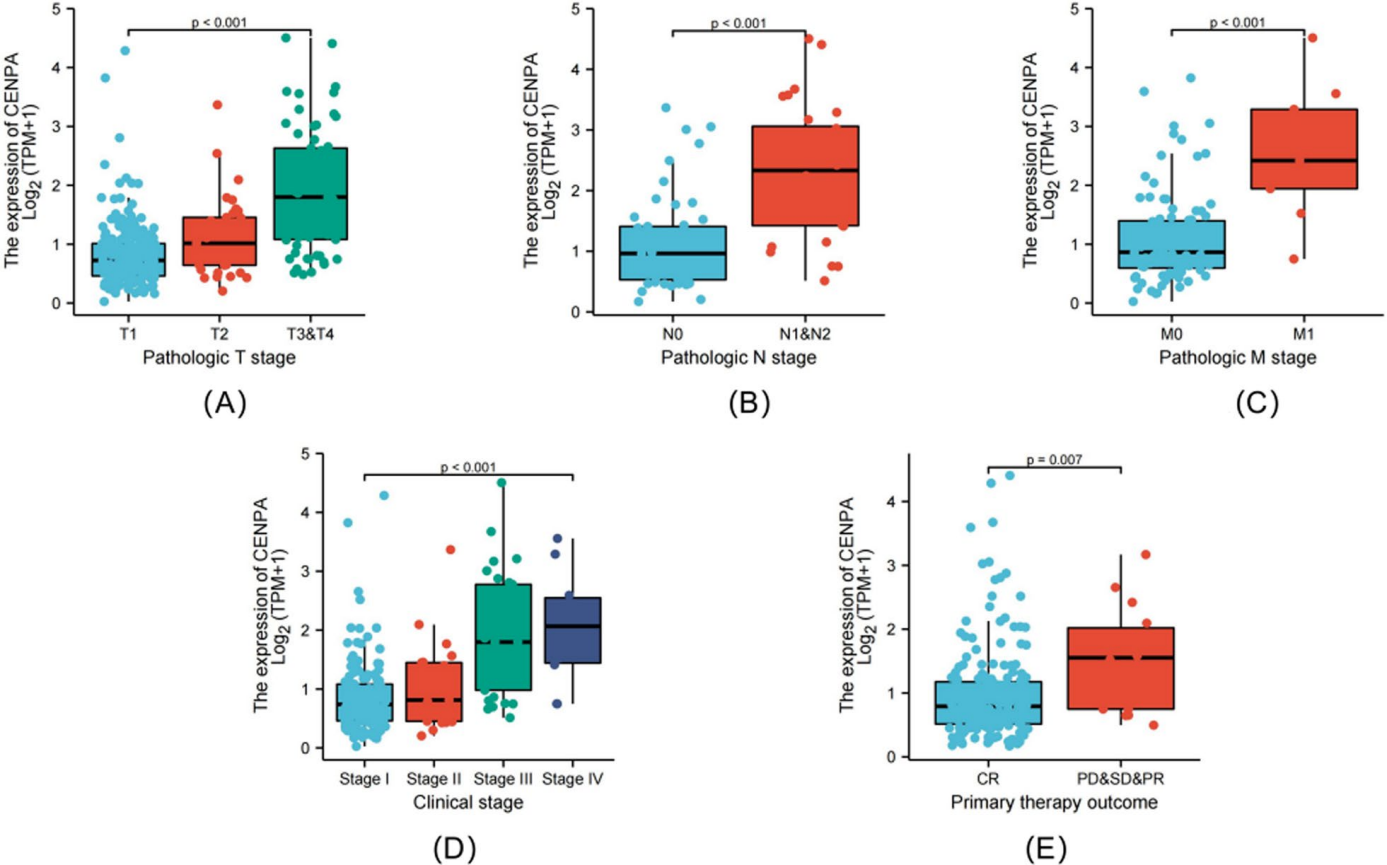
Association of CENPA expression with clinicopathologic characteristics. Pathological T stage (**A**); pathological N stage (**B**); pathological M stage (**C**); clinical stage (**D**) and primary therapy outcome (**E**)

**Fig. 3:**
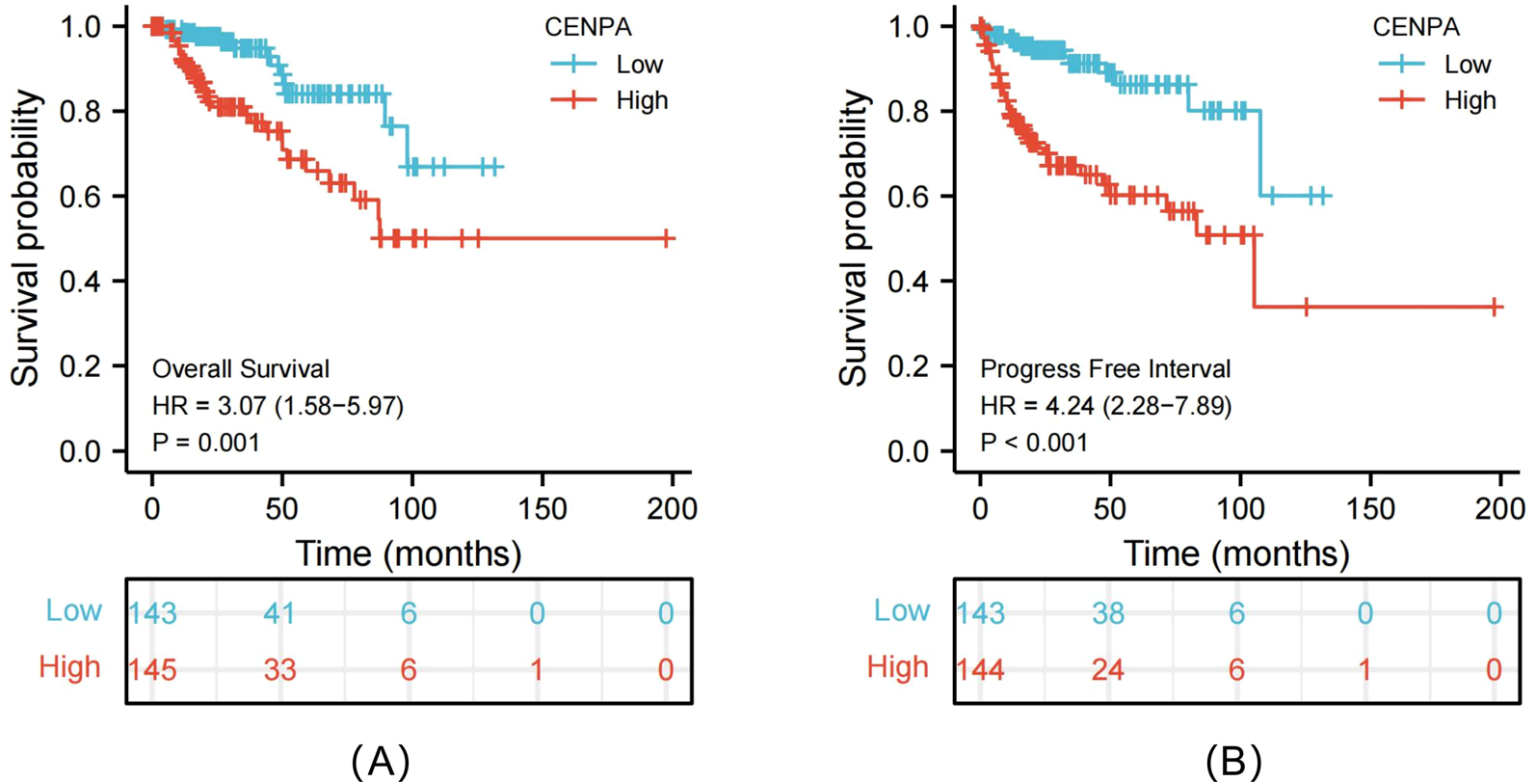
Kaplan–Meier survival curves comparing the high and low expression of CENPA in PRCC patients. Overall survival (OS) (**A**); Progression-free interval (PFS) (**B**)

As revealed by Kruskal–Wallis rank-sum test, CENPA expression was related to pathological T stage (*P* < 0.001), pathological N stage (*P* < 0.001), and pathological M stage (*P* < 0.001), clinical stage (*P* < 0.001), and primary treatment effects (*P* = 0.007) (Fig. 2A–E). Furthermore, KM survival analysis demonstrated that high CENPA expression was strongly associated with poor prognosis (*P* < 0.001) compared with low CENPA expression (Fig. 3A, B).

### Elevated CENPA expression predicts poor prognosis in different cancer stages

**Fig. 4:**
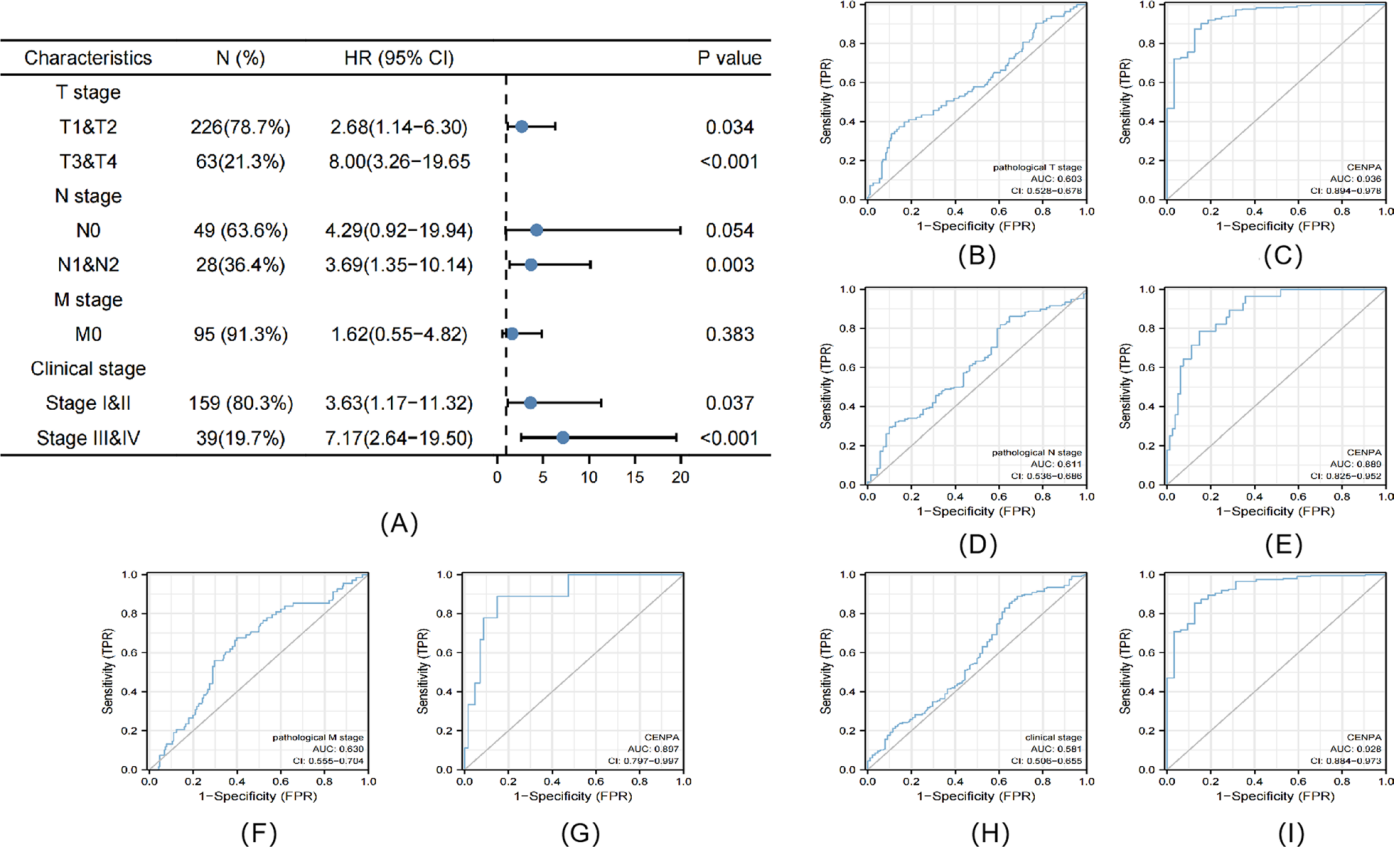
Multivariate survival analysis of OS concerning CENPA expression in patients of different subgroups according to cancer stage (**A**). ROC analysis of predictive accuracy in different subgroups, including pathological T stage (**B**), pathological T stage with CENPA expression (**C**), pathological N stage (**D**), pathological N stage with CENPA expression (**E**), pathological M stage (**F**), pathological M stage with CENPA expression (**G**), clinical stage (**H**) and clinical stage with CENPA expression (**I**)

As revealed by multivariate survival analysis, PRCC cases in the following tumor stages were associated with dismal survival, including clinical stage (I & II, *P* = 0.037, III & IV, *P* < 0.001), pathological T stage (T1 & T2, *P* = 0.034, T3 & T4, *P* < 0.001), pathological N stage (N0, *P* = 0.054, N1 & N2, *P* = 0.003), and pathological M stage (M0, *P* = 0.383) (Fig. 4A). Therefore, CENPA level affected PRCC prognosis in diverse clinical stages. At the same time, in order to investigate whether the expression of CENPA helps to improve the judgment of pathological TNM and clinical staging, we also performed ROC analysis for each subgroup. The AUC results showed that the predictive accuracy of pathological TNM and clinical stage was significantly improved after the inclusion of CENPA expression (T stage: 0.603–0.936; N stage: 0.611–0.889;M stage: 0.630–0.897; clinical stage: 0.581–0.928) (Fig. 4B–I).

### Role of CENPA in predicting PRCC survival under diverse clinicopathological characteristics

For further exploring CENPA-related mechanism within PRCC, this study assessed the relation of CENPA level with clinicopathological characteristics among PRCC cases through univariate Cox regression. More clinicopathological factors related to dismal prognosis included height, weight, primary treatment outcome, pathological T stage, and pathological N stage. For exploring survival-related factors, multivariate Cox regression was conducted on these factors. As a result, CENPA up-regulation was still a factor that independently predicted dismal OS, and other prognostic factors included height, weight, pathological T stage, pathological N stage and primary treatment outcome (Table 1).

**Table 1:** Association of clinicopathological characteristics with overall survival using univariate or multivariate Cox regression analysis

Characteristics	Total (N)	Univariate analysis		Multivariate analysis	
		Hazard ratio (95% CI)	*P* value	Hazard ratio (95% CI)	*P* value
Age	286				
< = 60	133	Reference			
> 60	153	0.944 (0.519–1.718)	0.851		
Gender	288				
Male	211	Reference			
Female	77	1.567 (0.813–3.021)	0.180		
Race	272				
White	205	Reference			
Black or African American	61	0.935 (0.413–2.116)	0.871		
Asian	6	5.966 (0.769–46.311)	0.088		
Height	212				
< = 170	87	Reference			
> 170	125	0.328 (0.155–0.696)	**0.004**	0.000 (0.000–0.000)	** < 0.001**
Weight	218				
< = 80	83	Reference			
> 80	135	0.413 (0.207–0.827)	**0.013**	0.000 (0.000–0.000)	** < 0.001**
Laterality	285				
Left	159	Reference			
Right	126	0.735 (0.392–1.376)	0.335		
Primary therapy outcome	199				
CR	185	Reference			
PD & SD & PR	14	7.157 (3.084–16.612)	** < 0.001**	0.000 (0.000–0.001)	** < 0.001**
Pathologic T stage	286				
T1 & T2	225	Reference			
T3 & T4	61	5.121 (2.790–9.397)	** < 0.001**	76,417,798,304,913,744.000 (7,520,202,477,862,371.000–776,532,269,571,339,520.000)	** < 0.001**
Pathologic N stage	77				
N0	49	Reference			
N1 & N2	28	5.003 (2.062–12.140)	** < 0.001**	0.000 (0.000–0.000)	** < 0.001**
Clinical stage	198				
Stage I & stage II	159	Reference			
Stage III & stage IV	39	9.489 (4.596–19.592)	** < 0.001**	1.000 (0.098–10.162)	1.000
CENPA	288				
Low	144	Reference			
High	144	3.102 (1.597–6.028)	** < 0.001**	282,613,493.616 (27,811,723.552–2,871,824,416.970)	** < 0.001**

Bold means characteristics with significantly different (*P* < 0.05)

*HR*, hazard ratio; *CI*, confidence interval; *PD*, progressive disease; *SD*, stable disease; *PR*, partial response.

### Construction and validation of the nomogram based on CENPA expression

**Fig. 5:**
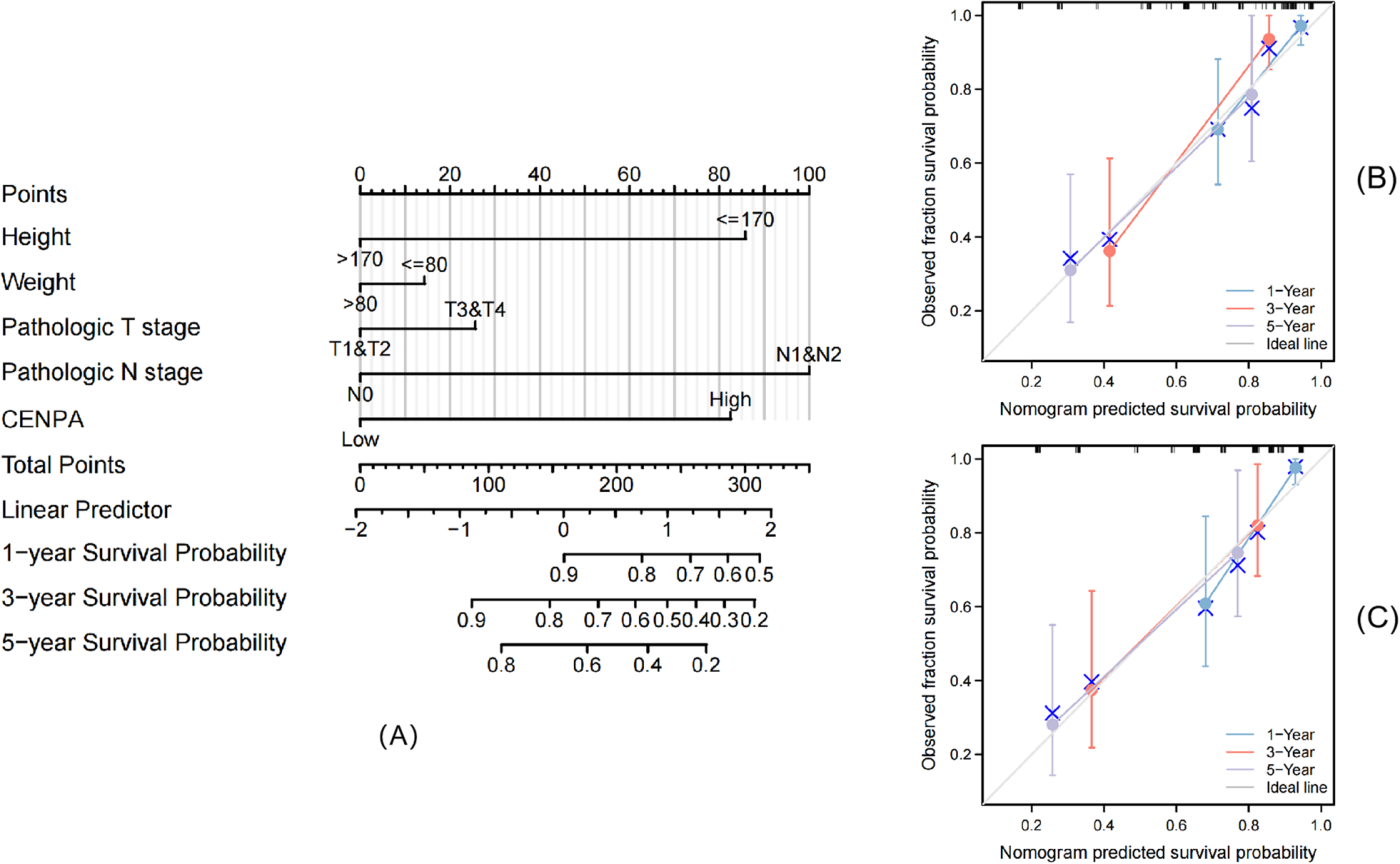
Relationship between CENPA and other clinical factors with OS. Nomogram for predicting the probability of 1-, 3-, and 5-year OS for PRCC patients in trial cohort (**A**). Calibration plot of the nomogram for predicting the OS likelihood in trial cohort (**B**) and validation cohort (**C**)

In trial cohort, the nomogram was established by integrating survival-related clinicopathological characteristics identified from multivariate analysis (height, weight, pathological T stage, pathological N stage and CENPA expression level), so as to offer a quantitative approach for clinicians (Fig. 5A) (Due to the small sample size, primary treatment outcome was not included in the nomogram production finally). These variables were incorporated into the nomograms based on multivariable Cox analysis using a point scale. We then accumulated the position of the variable and denoted it as a total score. Thereafter, we drew vertical lines from total-point axis along outcome axis to determine the 1-, 3- and 5-year survival probabilities for PRCC cases. After calculation, our nomogram had a C-index value being 0.822 (95% CI:0.779–0.865). Moreover, as observed from calibration graph, the deviation alignment line approached the ideal curve both in trial and validation cohorts, which indicated that those predicted results were well consistent with actual results (Fig. 5B, C). We also calculated the C-index, which included only other prognostic indicators after removing CENPA expression. The result was lower than our nomogram, which was 0.804 (95% CI:0.757–0.851). This indicated that CENPA expression can assist TNM stage to predict OS of PRCC to a certain extent. On the whole, our nomogram was a superior approach for predicting long-time PRCC prognosis to diverse prognostic factors.

### Determination of CENPA expression in the samples

**Fig. 6:**
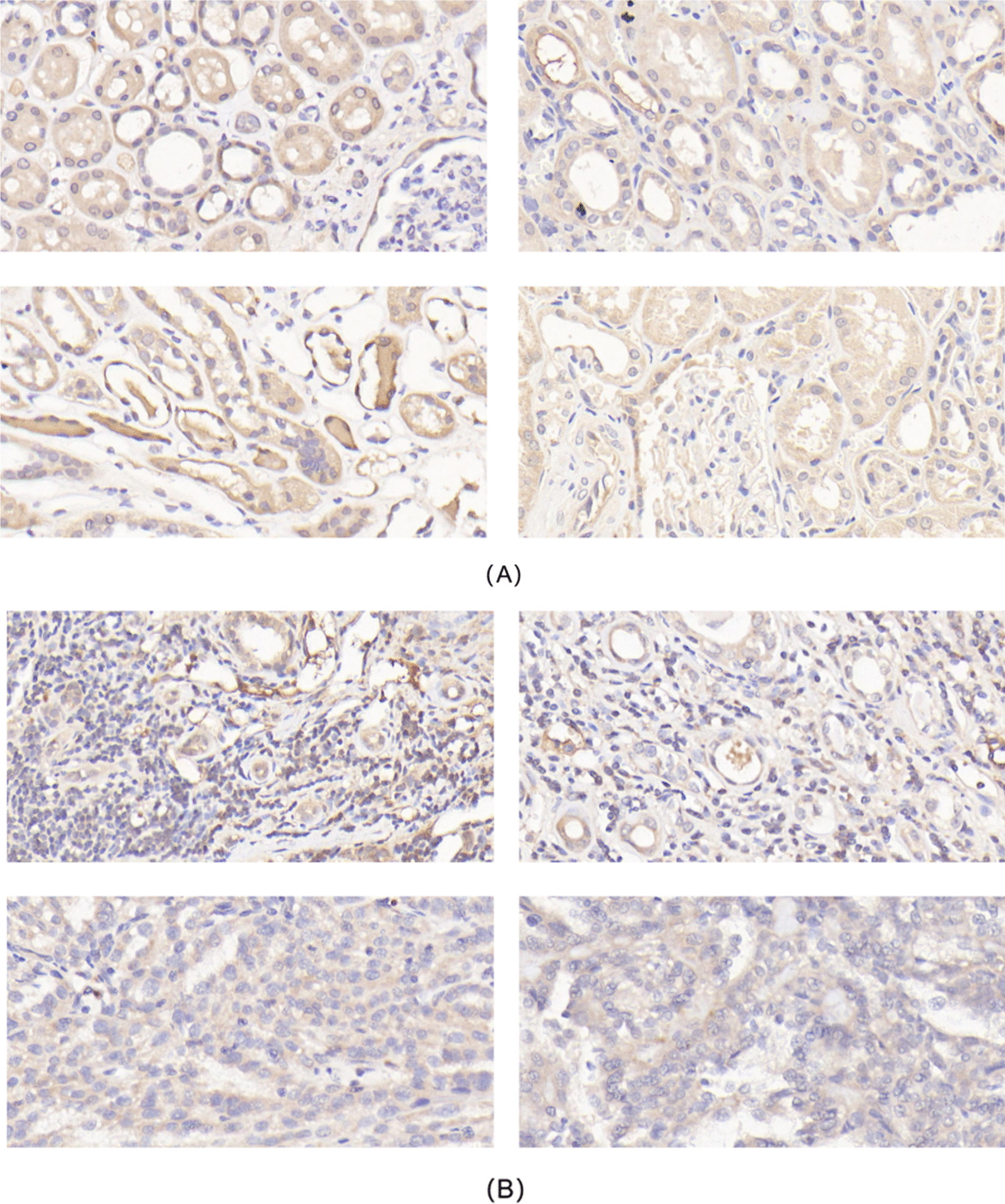
Expression of CENPA in the two gruops detected by IHC staining (× 400) **A**:Control group; **B**:PRCC group)

IHC staining was positive for the appearance of blue-purple particles. The score was rated in accordance with the percentage of positive cells and staining intensity. The scores of positive cell percentage were shown below, 0 (< 5%), 1 (5–25%), 2 (26–50%), 3 (51–75%), and 4 (> 75%), while the scores of staining intensity were as follows, 0 (no color or unclear), 1 (light purple), 2 (blue purple), and 3 (dark purple). Thereafter, the staining index (SI) was calculated as the product of positive cell percentage score and staining intensity score, which was expressed as negative for SI < 2 and positive for SI > 2. IHC results showed that the CENPA-positive rate was lower in control group than in PRCC group (Fig. 6A, B).

### Identification of DEGs between high and low CENPA expression groups

**Fig. 7:**
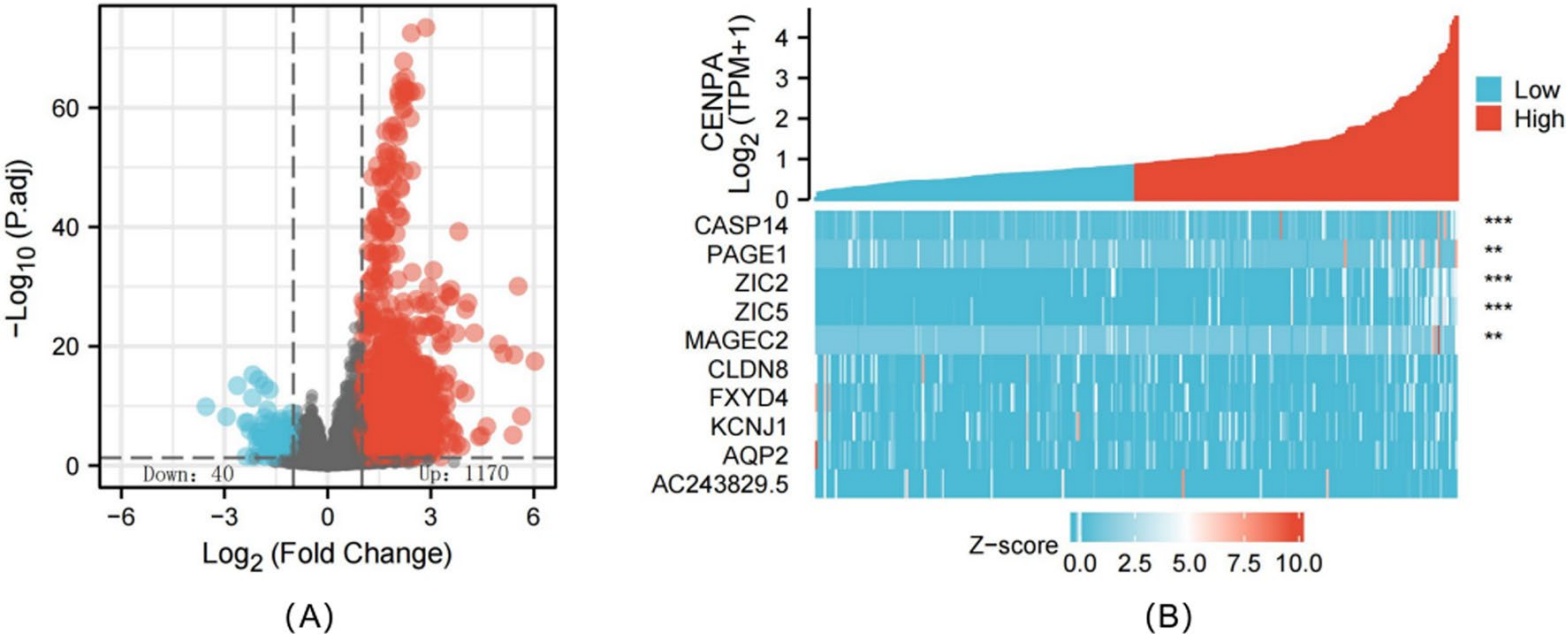
Differentially expressed genes between patients with high and low CENPA expression. Volcano plot of differentially expressed genes between the high and low CENPA expression groups. Normalized expression levels are shown in descending order from blue to red (**A**). Heatmap of the top ten significant differentially expressed genes between the high and low CENPA expression groups. Blue and red dots represent downregulated and upregulated genes, respectively (**B**)

We utilized the R software DSEeq2 package to analyze TCGA-derived data (adjusted *P* < 0.05 and |logFC|> 1.5) and discovered altogether 1210 DEGs between CENPA up-regulation and down-regulation groups, among which, 1170 showed up-regulation while 40 showed down-regulation in CENPA up-regulation group (Fig. 7A, B).

### Functional annotation and prediction of signaling pathways

**Fig. 8:**
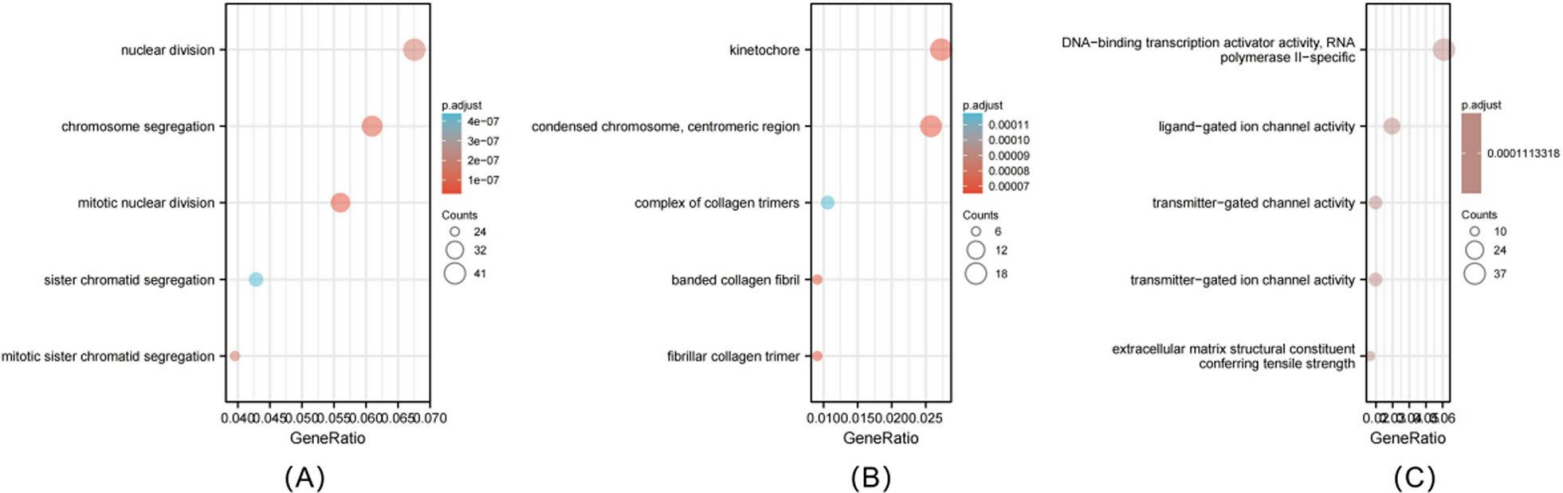
Enriched GO terms in the “biological process” category (**A**).Enriched GO terms in the “cellular component” category (**B**). Enriched GO terms in the “molecular function” category (**C**). Blue and red tones represent adjusted *P* values form 0.0–0.05, respectively, and different circle sizes represent the number of DEGs

For further understanding CENPA’s role in PRCC prognosis based on those 1210 DEGs discovered between CENPA up-regulation and down-regulation groups, we carried out GO annotation. Finally, we obtained 233 GO-biological process (GO-BP) terms, such as nuclear division, chromosome segregation, and mitotic nuclear division (Fig. 8A). The above findings indicated that abnormal CENPA level was related to nuclear division. In the meantime, we also discovered 18 GO-cellular component (GO-CC) terms, which confirmed that the abnormal CENPA expression was associated with kinetochore (Fig. 8B). In addition, the GO-molecular function (GO-MF) terms identified included the DNA-binding transcription activator activity, RNA polymerase II-specific was significantly enriched (Fig. 8C).

### The CENPA-associated signaling pathway based on GSEA

**Fig. 9:**
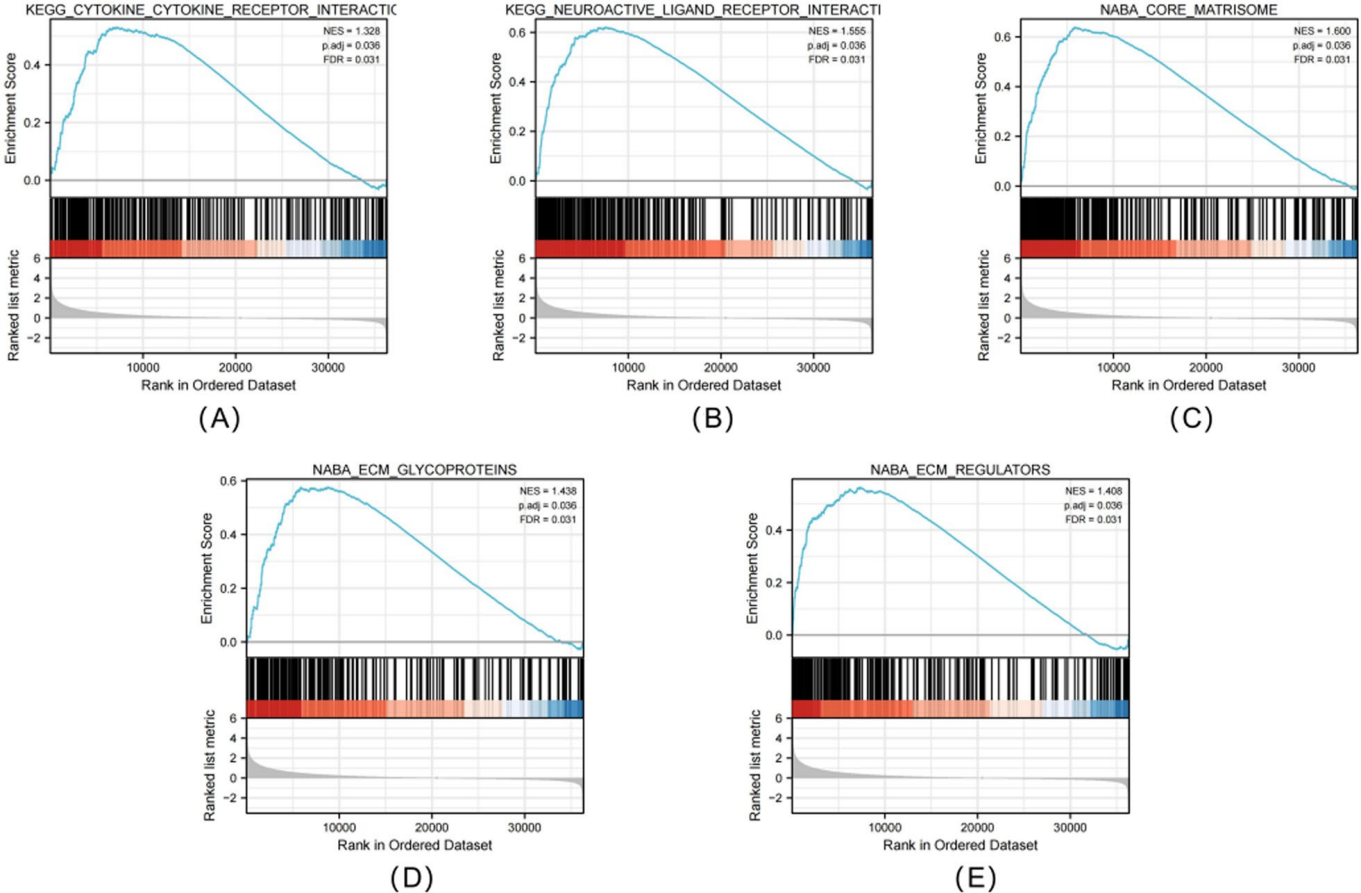
Enrichment plots from GESA. Several pathway were differentially enriched in PRCC patients according to high and low CENPA expression. Neuroactive ligand receptor interactions (**A**), cytokine receptor interactions (**B**), extracellular matrix regulators (**C**), extracellular matrix glycoproteins (**D**), nuclear matrisome (**E**). (ES, enrichment score; NES, normalized enrichment score; ADJ p-Val, adjusted *P*-value; FDR, false discovery rate).

Subsequently, signaling pathways related to PRCC were identified between high and low CENPA expression groups through GSEA using significant difference in MSigDB Collection (c2.all.v7.0) (adjusted *P* < 0.05, FDR < 0.25). Finally, 5 pathways, including neuroactive ligand receptor interactions, cytokine receptor interactions, extracellular matrix regulators, extracellular matrix glycoproteins and nuclear matrisome, were identified with significant differences between both groups (Fig. 9A–E).

### Correlation between CENPA expression and immune infiltration

**Fig. 10:**
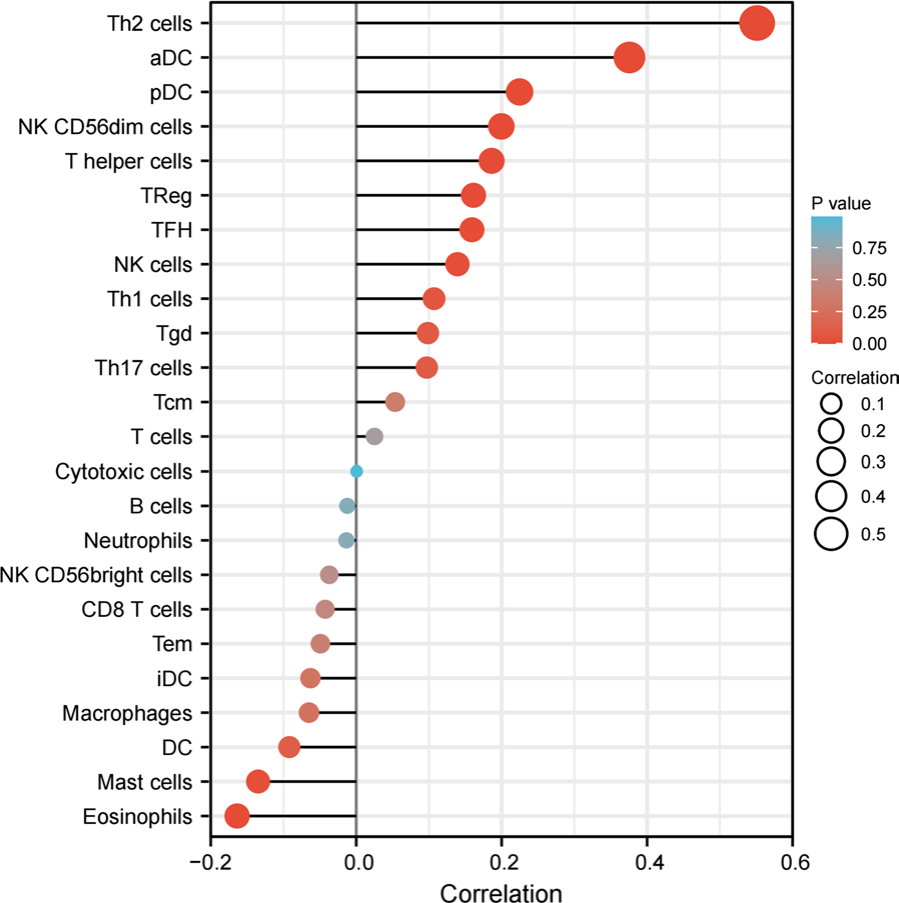
Correlations between the relative abundance of 24 immune cells and CENPA expression levels. The size of the dots represents the absolute Spearman’s correlation coefficient values

At last, this study examined the relation of CENPA level (TPM) with the enrichment levels of immune cells (obtained from ssGSEA) according to Spearman correlation analysis. As a result, CENPA level showed negative relation to enrichment levels of B cells, Cytotoxic cells, Nrutrophlis, NK CD56bright cells, CD8 T cells, TEM, iDC, Macrophages, DC, Mast cells, and Eosinophils. Moreover, CENPA level was positively associated with enrichment levels of Tcm, T cells, NK cells, Th17 cells, TReg, Th1 cells, TFH, T CD56dim cells, pDC, aDC, Th2 cells and Tgd (Fig. 10).

## Discussion

PRCC is the second most common type of renal cancer, however, there are few molecular mechanism studies and randomized clinical trials on PRCC due to the limited number of cases [3]. Recently, due to the rapid development of bioinformatics, the molecular mechanisms related to PRCC progression are being continuously explored. Although there are many clinical methods to treat PRCC, the patient prognosis remains poor. Therefore, it is of great necessity to identify the effective prognostic biomarkers to accurately predict PRCC prognosis and improve patient survival. With the continuous maturity of RNA sequencing and gene chip technology, the accuracy has been greatly improved, and the cancer-related big data network has been formed. In this regard, bioinformatics-based big data mining can easily find genes possibly related to the occurrence and development of PRCC. At present, massive studies are being conducted based on the bioinformatics prognostic analysis of PRCC [4–8].

The CENP family is considered as an important functional gene related to tumor development. High expression of centromere protein H (CENPH) is suggested to predict the poor prognosis of patients with tongue cancer [9], whereas overexpression of kinetocentromeric protein M (CENPM) can promote the occurrence of liver cancer [10], and overexpression of kinetocentromeric protein F (CENPF) can promote the development of NPC, GISTs and breast cancer (BC) [11]. CENPA in this study is a major component of the CENP family. As a centromere-specific variant, CENPA is identified as a key epigenetic marker for centromere recognition and reproduction, which has two main functions, including (1) providing essential conditions for centromere formation and maintenance, and (2) forming a platform for centromere assembly and mediating chromosome isolation [12]. This study again demonstrated these points by performing gene enrichment analysis on PRCC patient samples. As a result, abnormally high or low CENPA expression might disrupt genome integrity, resulting in chromosomal mis-polymerization and tumor occurrence. Previous studies have found that CENPA is highly expressed in more than 20 cancer tissues, including BC, colorectal cancer (CRC), lung adenocarcinoma (LUAD) and ovarian cancer [13–16]. Nonetheless, the effect of CENPA on predicting PRCC prognosis remains largely unclear. According to this study, CENPA served as the creditable factor to predict PRCC survival.

This work conducted bioinformatics analysis based on TCGA-derived high-throughput RNA-seq data for assessing CENPA’s effect on predicting PRCC survival. We found that CENPA expression within tumor tissue was higher than that in normal samples. Meanwhile, studies have discovered that overexpression of CENPA in cancer tissues is associated with poor clinicopathological factors, implying that CENPA most likely acts as an oncogene. Alternatively, high CENPA level was markedly related to dismal OS compared with low CENPA expression. Therefore, we speculated that CENPA was the biomarker for PRCC. At the same time, compared with other prognostic biomarkers of PRCC currently being explored, CENPA has its own unique ability. Its expression level can not only improve the predictive accuracy of traditional pathological TNM and clinical staging, but also help pathological TNM staging to better predict the OS of PRCC patients. For better investigating the source of these advantages, the TCGA-derived data based on GSEA were utilized to analyze CENPA’s effect on PRCC. As a result, associated genes regulated by neuroactive ligand receptor interactions, cytokine receptor interactions, extracellular matrix regulators, extracellular matrix glycoproteins and nuclear matrisome were differentially enriched in the PRCC phenotype with high CENPA expression. However, whether CENPA exerts the synergistic or complementary effects with these pathways remains unclear, and detailed regulatory networks are unavailable for the time being. Moreover, CENPA was related to diverse complicated crosswalk related to additional genes. Consequently, mechanisms related to the above interactions should be further investigated in details.

Tumor microenvironment (TME) has been considered as an important interface that mediates tumor cells’ physiological responses, while tumor-infiltrating immune cells (TIICs) represent a crucial component in TME, and their levels and composition have been identified to be related to tumor survival [17, 18]. On this basis, this work analyzed the relation of CENPA level with immune infiltration degrees within PRCC (Fig. 9). It suggested that the increased CENPA expression was inversely related to the abundance levels of B cells and CD8 + T cells in PRCC. Besides, it has been shown that the low levels of B cells and CD8 + T cells in tumor tissues are associated with the prognosis of cancer patients [19–21], containing the poor prognosis of PRCC. Several other studies report that the high levels of tumor-infiltrating Tregs in BC, HCC, lung cancer, gastric cancer, and ovarian cancer were significantly associated with the poorer prognosis [22–26].Therefore, we reasoned that CENPA might affect patient prognosis by modulating the immune infiltration in PRCC.

Our study provides new insights into the correlation between CENPA and PRCC, but there are some limitations to be noted. First, we only evaluated one TCGA dataset, which might cause sample bias. Secondly, the sample size should be further expanded to increase the credibility of our results. Thirdly, further experimental validation is warranted to elucidate the in-vitro and in-vitro biological functions of CENPA.

## Conclusion

This study reveals for the first time the prognostic value of CENPA in PRCC. Our results suggest that CENPA has the potential as a biomarker to predict treatment outcome and prognosis of PRCC patients. However, further experiments are still needed to validate the biological effects and underlying mechanisms of CENPA.

## Data Availability

The material supporting the conclusion of this review has been included within the article.
